# Energy of Intramolecular Hydrogen Bonding in *ortho*-Hydroxybenzaldehydes, Phenones and Quinones. Transfer of Aromaticity from *ipso*-Benzene Ring to the Enol System(s)

**DOI:** 10.3390/molecules22030481

**Published:** 2017-03-18

**Authors:** Danuta Rusinska-Roszak

**Affiliations:** Institute of Chemical Technology and Engineering, Poznan University of Technology, ul. Berdychowo 4, 60-965 Poznan, Poland; danuta.rusinska-roszak@put.poznan.pl

**Keywords:** HOMA, intramolecular hydrogen bond, MTA, RAHB

## Abstract

Intramolecular hydrogen bonding (HB) is one of the most studied noncovalent interactions of molecules. Many physical, spectral, and topological properties of compounds are under the influence of HB, and there are many parameters used to notice and to describe these changes. Hitherto, no general method of measurement of the energy of intramolecular hydrogen bond (E_HB_) has been put into effect. We propose the molecular tailoring approach (MTA) for E_HB_ calculation, modified to apply it to Ar-O-H∙∙∙O=C systems. The method, based on quantum calculations, was checked earlier for hydroxycarbonyl-saturated compounds, and for structures with resonance-assisted hydrogen bonding (RAHB). For phenolic compounds, the accuracy, repeatability, and applicability of the method is now confirmed for nearly 140 structures. For each structure its aromaticity HOMA indices were calculated for the central (*ipso*) ring and for the quasiaromatic rings given by intramolecular HB. The comparison of calculated HB energies and values of estimated aromaticity indices allowed us to observe, in some substituted phenols and quinones, the phenomenon of transfer of aromaticity from the *ipso*-ring to the H-bonded ring via the effect of electron delocalization.

## 1. Introduction

Noncovalent interactions, such as hydrogen bonding, play an important role in supramolecular chemistry, drug-receptor interactions, drug design in chemical and biological processes, including molecular recognition, and the bioactivity of macromolecules [1,2,3]. Hydrogen bonds (HBs) allow the definition of the crystal packing of many organic and organometallic structures, are the source of interesting properties of associated liquids, and can give valuable insights on the solubility of molecules. For almost one hundred years, different types of hydrogen bonding have been described, classified, measured, and compared, often providing an unquestionable explanation of numerous biological processes. Hydrogen bonds can be broadly classified as very strong, strong, medium, and weak, as cooperative and anti-cooperative, chelated and bifurcated, classic and unconventional, intermolecular and intramolecular, and resonance-assisted (RAHB) [4,5] and charge-assisted ((+)CAHB and (−)CAHB) [6]. Classic types of HB occur between a hydrogen atom covalently bound with a strong electronegative atom (such as O, N, Cl, or F) and another electronegative atom, which plays the role of the hydrogen acceptor. In unconventional HBs, the C-H group [7,8,9] acts as a donor, while anions and π-electrons from unsaturated bonds or aromatic rings act as hydrogen acceptors [6,10].

The strength of a hydrogen bonding can be modified by the presence of substituents, molecule configuration, and conformation possibilities, and induce different effects to electron delocalization in hydrogen-bonded systems. Geometrical, physical, topological, and spectral parameters are used [11,12,13] for the estimation of the strength of intermolecular bonding with good accuracy. When both the donor and the acceptor fragments belong to the same molecule, such estimations become more difficult.

The most commonly used method to estimate the energy of the intramolecular HBs is based on the *cis-trans* (or *syn-anti*) comparative analysis method [1,2,4,6]. For two conformers that essentially differ by the presence of one intramolecular HB, a *trans*-conformer can be built by rotation of the hydrogen atom up to 180 degrees, with the optimization of such a prepared structure, or without. The energy of hydrogen bonding is estimated as the difference between the energies of both conformers.

The isodesmic reactions appear to provide more reasonable results than the *cis-trans* approach when used to evaluate the intramolecular interaction energy [14,15,16]. In isodesmic reactions [16] the number and types of bonds are conserved on the reactant and product sides of the reaction. The isodesmic method was used for the estimation of intramolecular HB energies based on the assumption that the total molecular energy can be partitioned into energies of chemically recognizable fragments [17,18]. This method is advocated for systems with one HB, but it is not recommended for the estimation of the single intramolecular H-bond energy in polyhydroxy systems [17].

The interatomic interactions, such as hydrogen bonds, can be described and classified by the electron density ρ at the (3,–1) bond critical points (BCP) [19,20,21,22]. The ring critical points (RCP) found in the intramolecular HB region, also play a role in the estimation of interaction by reinforcing the binding for intermolecular bifurcated HBs [23] and for compounds with RAHB [24,25], even in two-ring (chelated intramolecular HB) systems. The electron density and other Atoms In Molecules (AIM) parameters at the RCP correlate with aromaticity indices and can be used for the estimation of the electron delocalization effect in aromatic and quasiaromatic ring systems [10,26]. Over the last two decades, the theoretical analysis of electron density topology (AIM theory) has been the most widely used method for the investigation of hydrogen bond systems by the electron density at the bond critical point ρ_BCP_, its Laplacian ∇^2^ρ_BCP_ value and the potential energy density V_BCP_, which are occasionally treated as universal descriptors of the hydrogen bond strength [10,27,28]. The equation formulated by Espinosa [27] $E_{HB}=\frac{1}{2}V_{BCP}$ allows the calculation of the HB energy based on the potential energy density in its bond critical point (V_BCP_). Recently Afonin [28] introduced modifications to this equation using different linear regression coefficients in order to obtain a better correlation.

In 1994, Gadre [29] introduced the Molecular Tailoring Approach (MTA) method of ab initio quality computation of various electron properties of (large) supermolecules, which involves construction of the density matrix of the supermolecule from block matrices of smaller fragments, each representing a part of the supermolecule. This method was primarily drawn up for calculation of properties of silicious zeolite-clusters [29], and further for various biologically active systems as taxol, γ-cyclodextrin, α-tocopherol, and other large organic or inorganic crystalline substances [29]. Next, Deshmukh [21] used this method for systems containing multiple O-H∙∙∙OH intramolecular hydrogen bonds. Their energy was calculated by the fragmentation approach: the original optimized molecule was cut into three overlapping fragments, which are obtained by replacing the OH groups with the hydrogen atom, without optimization, to avoid conformational changes in it. It was shown that MTA yields more reliable H-bond energy values than the isodesmic method, and can be easily applied to any complicated polyhydroxy H-bonded systems.

In the study carried out by Deshmukh et al. [17], it was demonstrated that the estimated MTA values are consistent with the corresponding H-bond lengths, but the isodesmic/homodesmic reaction approach is not a good enough method for the estimation of H-bond energy for multiple H-bonded intramolecular systems. In contrast, the molecular tailoring approach yields more reliable H-bond energy values and can be easily applied to any complicated H-bonded systems with large numbers of OH∙∙∙OH interactions. This offers several interesting possibilities for exploring intramolecular interactions in large biomolecules. The typical error involved in the calculation of is quite small (~0.5 kcal/mol for polyalcohols) [21].

The method was developed by us for saturated hydroxycarbonyl compounds with six-, seven-, and eight-membered ring of intramolecular hydrogen bonds [30], and for many structures with various types of RAHB [25]. In case of the first group of compounds, the examination of 140 examples confirmed that the method gives reproducible results, precise and congruent with some of the aforementioned parameters, such as the length of HB, length of the O-H covalent bond, distance of the O∙∙∙O, IR frequency of the O-H group, chemical shift of the H-bonded proton, electron density, and its Laplacian (∇^2^ρ_BCP_) at the bond critical point. In case of the second group (RAHB), the fair agreement of calculated dependences was observed for the length and angle of the HB, covalent O-H bond and the O∙∙∙O distance, O-H frequency, and the electron density in the critical bond and ring critical bond. The HB angle, δ_H_ and ∇^2^ρ_BCP_ of intramolecular resonance-assisted hydrogen bond values were not useful for estimating of HB strength. Peculiar exceptions may be observed even in the case of good relationships, which exclude the use of the aforementioned parameters, but are explicable from a structural point of view.

In our previous reports [25,30] the aromatic hydroxycompounds, i.e., phenols were not interpreted, because it was found that they represent a different type of intramolecular hydrogen bond. Aromaticity is a collective phenomenon of π-conjugation, sensitive to different external effects, which also exerts mutual influence. Such structures have the same phenolic group as a hydrogen bond donor, and constitute a considerable part of naturally-occurring substances, which are important in biochemical processes, as well as in medicinal and pharmaceutical applications. For example some flavonoids exhibit anticancer, anti-inflammatory, and anti-oxidant properties, acting as free radical scavengers, due to the dissociation of the OH bond [31]. Therefore, it is important to recognize the role played by the HB in stabilizing radicals and the anionic species in phenols, for example, in acylphloroglucinols [32,33]. Usinic acid [34] produced by lichens acts as an effective antibiotic and scytalone dehydratase is crucial for the fungal melanin biosynthetic pathway [35]. Some quinones (lapachol, caryopteron) [36] exhibit important cytotoxic activity against cancer cells, naphthazarin [37] acts as an antibacterial agent, apoptosis inducer, antineoplastic agent, and the most common application of anthraquinones remains the dyeing of both natural and synthetic fibres [38].

The strength of the hydrogen bond in saturated hydroxycompounds was estimated at 1.4–7 kcal/mol, additional unsaturated, but not conjugated, elements increased it to 13.7 kcal/mol [30] while, in the case of structures with RAHB, the energy of the hydrogen bond (E_HB_) was calculated in the range of 8.2 to 23.6 kcal/mol [25] with 14.5 kcal/mol for the representative enol of malonaldehyde. Due to the additional external four- and five-membered stiffening rings, this value may be weakened and is sensitive to the changes resulting from various substitution.

In this study, mono- , di-, and triphenols substituted at the *ortho*- position by various functional groups containing carbonyl, and additionally substituted by different groups donating or withdrawing electrons (Table 1), were examined. The study includes hydroxyquinones and anthraquinones, as they exhibit the same relationships between energy and other characteristics of the hydrogen bonding (Table 2 and Table 3). Some natural or more complicated structures, in which the phenolic group is engaged in intramolecular HB with the carbonyl group, were described separately. The structures with the phenolic group involved in seven- or eight-membered HB ring, which are characterized by low E_HB_, do not comply with structural conditions and are presented in the Supplementary Materials. The term energy of hydrogen bonding (E_HB_) has been used throughout the article to express the difference between energies of two molecules: one which is stabilized by hydrogen bonding, and another in which “the internal HB is broken without causing other structural or electronic changes” [11].

Note that substituted, condensed polycyclic hydrocarbon systems which contain hydrogen bonded molecules are excluded from this work, due to their dependence on the position of -OH and –C=O substitution [39,40].

## 2. Computational Methods

For estimating the O-H∙∙∙O=C intramolecular hydrogen bond energy, a systematic fragmentation of each optimized molecule was carried out, using modified Deshmukh’s [21] methodology, which consists of comparing the energies of the fragments of a molecule, in which the atoms of the donor, acceptor, and both groups forming the hydrogen bond are successively removed. This allowed avoiding contact between both groups in any such built structures.

**Scheme 1:**
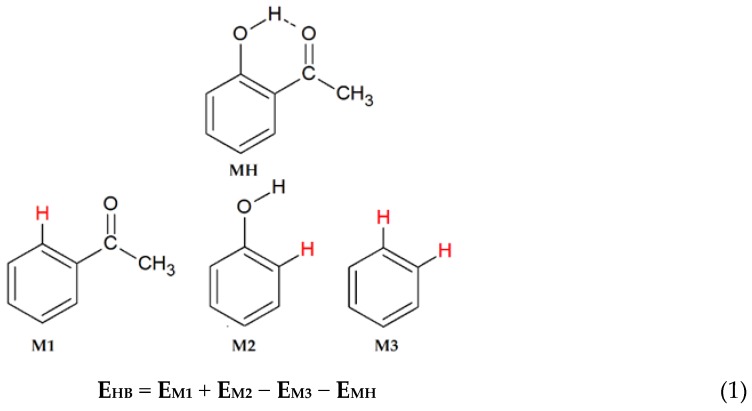
General principles of molecular tailoring approach (MTA) fragmentation.

Each functional group participating in intramolecular hydrogen bonding was replaced by an inserted hydrogen atom, as in [25,30]: the valences of the cut regions (atoms) were satisfied by the addition of the hydrogen atoms at appropriate directions and arbitrary constraint distances equal to 1.1 Å [21]. It is very important that the hydrogen atom replaces only single but not multiple bonds, and that after this operation the distance between both added hydrogen atoms is greater than 2.2 Å (double the van der Waals radius) to avoid steric hindrance. We propose the fragmentation of the hydrogen-bonded structure in such a manner that the C=O group is removed with the part of the molecule “behind” C_α_, C_β_, or C_γ_ at the site opposite to the OH group (examples are presented in Supplementary Materials, Figure S1). The energies of such parts (Scheme 1) were calculated without optimization at the MP2(full)/6-311++G(2d,2p) level and used to calculate the strengths of hydrogen bonds according the Equation (1) given in Scheme 1.

All candidate structures were first optimized by the B3LYP density function [41,42] of the B3LYP/6-311++G(d,p) basis set [21] using the Gaussian 09 [43] program package, as described previously [25,30], due to the fact that this method provided credible results in the past [44] when dealing with H-bonded systems. In the next step the structures were checked by the vibrational analysis at this level and were found to represent the true energy minima. The calculated ν_OH_ and ν_C=O_ frequencies were identified with the GaussView 5.0.9 package, without correction for the zero-point energies. At the same level, the absolute proton shielding for each structure and TMS (tetramethylsilane standard) were obtained using the gauge-including atomic orbital (GIAO) method [45]. The SCF GIAO magnetic isotropic shielding tensors of H-bonded hydrogen atoms were used to calculate the chemical shifts (δ_H_). Afterwards, the electron correlation was included via the Møller-Plesset treatment of the second order (MP2), in which each structure was finally optimized with MP2(FC)/6-311++G(2d,2p) and its energy was specified with MP2(full)/6-311++G(2d,2p) calculations. The topological properties of the electron density at the bond critical points (BCPs) were characterized using the Bader Atoms In Molecules methodology (AIM) with the AIM2000 [46] and AIMAll [47] program packages for every fully-optimized geometry. The AIM analyses of the wavefunction have been conducted on true MP2 wavefunctions (using “natural orbitals” of the MP2 first-order total density matrix). Similarly to a previous report [25,30], the electron density ρ_BCP_ at the (3,–1) bond critical point, its Laplacian ∇^2^ρ_BCP_ (for the BCP between the hydrogen atom of the OH donor group, and the oxygen atom of the O=C acceptor group), potential energy density V_BCP_, and the electron density ρ_RCP_ (at the ring critical point in the centre of the newly-formed six-membered ring, which includes the hydrogen bonded atoms, further named as the “HB-ring”) were selected to describe the nature of the hydrogen bonding.

Aside from the above-mentioned parameters used to describe the electron distribution in the intramolecular hydrogen bonds, additional geometry-dependent indices of the aromaticity proved useful in the case of substituted phenols. The extra ring formed by substituents interacting through the hydrogen bond in phenols affect the strength of the HB and the local aromaticity of the aromatic ring [48,49]. The phenolic ring, named *ipso*-ring, can be described by the Harmonic Oscillator Model of Aromatic stabilization (HOMA) index [12,50,51,52] defined by Krygowski’s equation (Equation (2)):

(2) $$\mathrm{HOMA}=1-\frac{\alpha }{n}\sum^{\text{​}}{\left(R_{opt}-R_{i}\right)}^{2}$$

where the number of bonds taken into the summation n = 6; α = 257.7, R_opt_ = 1.388 Å. and R_i_ stands for the running bond length.

The interaction between the ring and the hydrogen bond is regarded to and named as *quasi*-aromatic, and can be described by the quasiHOMA index, which was calculated by Equation (2), where the number of all bonds taken into the summation n = 4, i.e., for C-O and C=O bonds α = 157.38, R_opt_ = 1.265, and for C-C bonds α = 257.7, R_opt_ = 1.388 Å, and R_i_ stands for C-O, C=O, C-C, and C_ar_-C_ar_ bond lengths.

The strength of the HB and its relation with the *π*-electron delocalization within the *ipso*-ring is discussed, and analysis of the aromaticity of the *quasi*-ring is performed in relation to the strength of the intramolecular HB that is formed.

## 3. Results

The geometry of each intramolecular hydrogen bond for 140 fully-optimized structures was described using four geometrical parameters: length of the O-H covalent bond (d_OH_), the distance of the HB as H∙∙∙O (r_HB_), the distance between both the proton donor and proton acceptor oxygen atoms as O∙∙∙O (d_O∙∙∙O_), and the angle of the HB as O-H∙∙∙O (φ_HB_). The complementary data, including the calculated frequency of O-H and C=O stretching, the hydrogen chemical shift, and HOMA indices, including the AIM characteristics of the analysed structures, are collected in Table S1 (Supplementary Materials). Only the diagrams which show significant correlations between the listed parameters and the calculated E_HB_ are included at the end of this study (*vide infra* Figure 6A–J).

Table 1 contains the structural parameters of stable conformers of phenolic intramolecular hydrogen bonding for unsubstituted *o*-hydroxycarbonyl compounds, substituted *o*-hydroxyaldehydes, and substituted *o*-hydroxyketones and acids. The last column contains data regarding the related strength of HB cited in the literature. Note that the E_HB_ are given as positive values.

The last column of Table 1 presents literature E_HB_ values calculated by different methods which are generally inconsistent. The values obtained with the close-open method [44,54,59] seem to be overestimated, even up to two-times, whereas the values obtained with the homodesmic [53], as well as the potential energy density, methods [28] are similar to those presented in this study.

In the first part of Table 1, aside from salicylaldehyde **1**, acetophenone **2**, benzophenone **3**, and salicylic acid **4**, a number of acid derivatives are presented: esters, amides, sulphides, or halides.

The vast majority of intramolecular HB energy values calculated for phenols listed in Table 1 range from 5.4 to 10 kcal/mol. The weakest HBs are observed in the case of acid halides **14**, **15**, and **16,** whereas the strongest HBs occur in tri-substituted structures **44** and **70**, as well as anions **47** and 48, in which the decreased HOMA value indicates a loss of aromaticity in the *ipso* ring. The quasiHOMA index values are more diverse: from approximately zero to 0.427, or even 0.494 in the case of structure **44**, and confirm the high influence of electron donor properties, as well as the position of substitution. The notable decrease of aromaticity in the quasiaromatic ring (HB-ring) is caused by substituents, which may conjugate with the carbonyl group (**8**–**11**).

**Figure 1:**
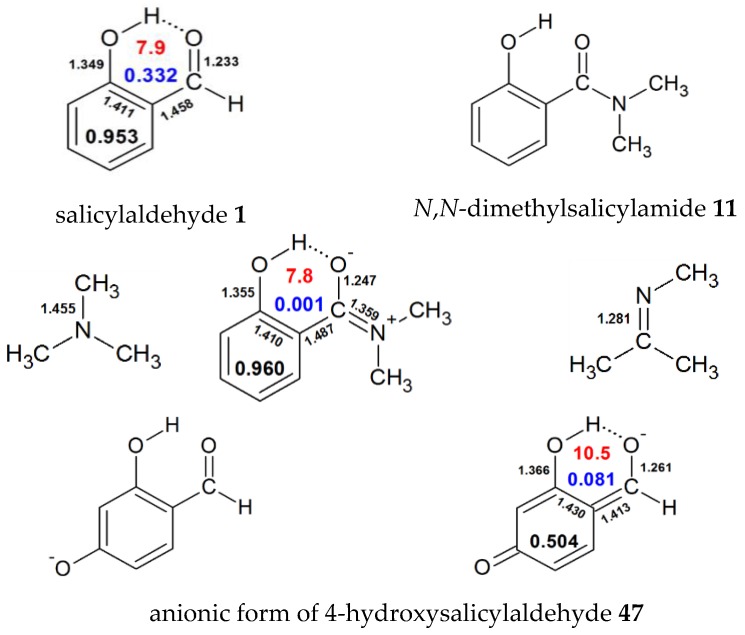
The E_HB_ (in red) and selected geometric parameters of salicylaldehyde **1**, *N*,*N*-dimethylsalicylamide **11** and 2-hydroxy-4-hydrylidylbenzaldehyde **47** as well as their essential resonance structures; HOMA and quasiHOMA (in blue) values were given in relevant rings.

The explanation is shown in Figure 1, in which energetic and geometric parameters of salicylaldehyde 1 are compared with *N*,*N*-dimethylsalicylamide **11**. The elongation of C=O, C_ar_-C(=O) and C-O bonds is a result of participation of free electrons of amide nitrogen atoms in carbonyl resonance. Comparison of the obtained values of bond length, C-N-C angle, and Wiberg bond order [65] for the C-N bond in trimethylamine (1.458 Å, 110.1, 0.996) and trimethylimine (1.281 Å, 118.8, 1.862) (optimized as each of the analysed compounds) with these values obtained for structure **11** (1.359 Å, 117.5, 1.173) confirms the proposed resonance structure which includes the sp^2^ hybridized nitrogen atom.

Interestingly, when the -CN (structure **17**) or -NO_2_ (structure **18**) groups (the only substituents without a conjugated lone electron pair) are linked as the R_1_ group, the hydrogen bond is weaker and quasiHOMA is approximately as high as in salicylaldehyde **1** (Table 1).

In the group of substituted salicylaldehydes, the energies of HB are approximately at the same range (7.5–8.9 kcal/mol), with a few exceptions (2,4,6-trihydroxybenzaldehyde **41**—9.4 kcal/mol; the mono anionic form of 2,4-dihydroxybenzaldehyde **47**—10.5 kcal/mol; triformylphloroglucinol **44**—11.3 kcal/mol). The quasiHOMA index for most compounds in this group (0.29–0.42) resembles the value of the model compound 1. Note that the presence of the oxido (phenolic anion) group in the R­_4_ position (*para*- to the carbonyl) in the anionic structure **47** causes almost total dearomatisation of the HB-ring, and a strong decrease of the HOMA index of the *ipso*-ring (to ca. 0.5). This may be caused by the resonance of extra electrons, which impairs aromaticity and creates the (−)CAHB character of HB. This explanation is evident in Figure 1, in which the stronger HB arises with the elongation of C-O, as well as C=O and shortening of C_ar_-C bonds (relative to salicylaldehyde reference **1**).

In the substituted hydroxyketones group in Table 1, the strength of HB in the case of hydroxyketones is higher (average E_HB_ = 8.57 kcal/mol) compared to related aldehydes. The halogen substitution at the R_3_ position of salicyl acid **4** has no influence on the energy of intramolecular HB in structures **71**, **72**, and **73** [61].

**Figure 2:**
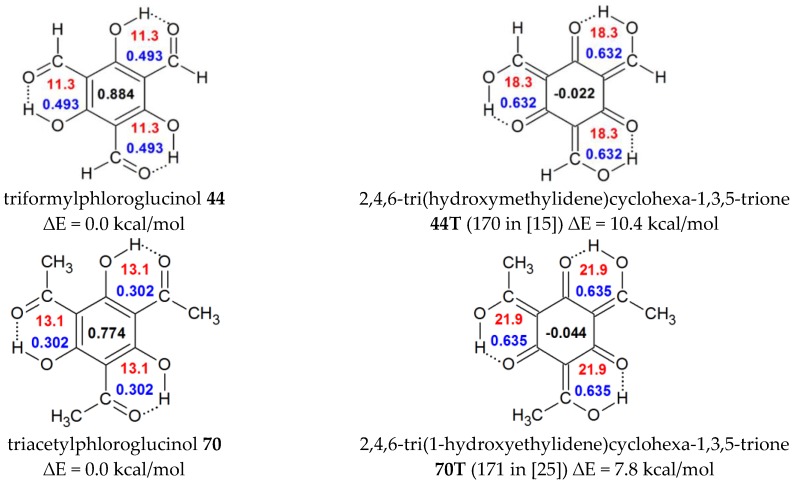
The transfer of aromaticity in triformyloglucinol **44** and triacetylphloroglucinol **70** from the *ipso*-ring to the enolic system. Values of quasiHOMA (in blue) and E_HB_ (in red) were given in the centre of the HB-ring, whereas the HOMA value was given in the *ipso*-ring.

In the case of triformylphloroglucinol **44** a remarkable reduction of the aromaticity of *ipso*-ring may be observed, which is also likely in its corresponding triacetyl derivative **70**. The tautomeric forms of both compounds **44T** and **70T** were presented in a previous study [25] concerning RAHB. The tautomers **44T** and **70T** arise from ketoforms through the displacement of three protons with the allied changes in geometry and rearrangement of π-electrons in the system—from the *ipso*-ring to the outside. The results are shown in Figure 2.

The structures of formyl- and acetyl- phloroglucinols **44** and **70** are much more stable compared to their enolic analogues **44T** and **70T**, with the difference of ∆E values at 10.4 and 7.8 kcal/mol, respectively. In less stable RAHB structures, the HOMA indices of *ipso*-rings fall to near zero values, and the quasiHOMA indices of the rings involved with HBs increase almost twice, with a significant increase of the HB’s strength. These enolic structures resembles the triphenylene, in Clar’s aromatic π-sextet rule for polycyclic aromatic hydrocarbons (PAHs), considered as more stable and more aromatic than the other rings in benzenoid species. Therefore, the internal *ipso*-ring is often named an “empty” ring having six π-electrons [66,67] and the observed changes are named as transfer of aromaticity.

Table 2 contains results obtained for hydroxynaphthoquinones. Similarly, Table 3 reports hydrogen bonding in selected hydroxyanthraquinones; however, a number of more complex structures of biological importance are presented separately in Table 4. Some of the calculated energies of HB were compared with the previously published values [34,37,58,68,69,70,71,72].

In terms of energy, the hydroxynaphthoquinones are similar to monohydroxyketones **2** or **3** (E_HB_ of approximately 8.5 kcal/mol), as well as for HOMA indices, even with additional hydroxyl-, aryl-, or alkyl- substituent. In the case of naphtazarin **83**, a dihydroxynaphtoquinone, and the remaining quinones stabilized by two intramolecular HB, their E_HB_ increased beyond 10 kcal/mol, because of the effect of extra OH substitution, with free electrons, which participate in the whole aromatic system.

The substitution by one, two, three, or four chlorine atoms revealed the sensitivity of the applied MTA method of calculation to asymmetrical substitution. However, the differences among E_HB_ values associated with the position of substitution and number of Cl substituents are low.

**Figure 3:**
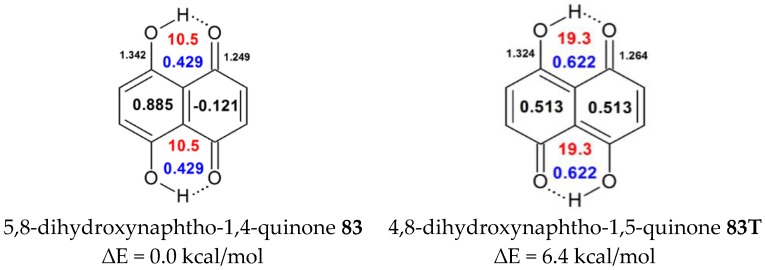
The transfer of aromaticity from *ipso*-ring to the enolic system in naphthazarin. Values of quasiHOMA (in blue) and E_HB_ (in red) were given in the centre of the hydrogen bonded ring, whereas the HOMA value was given in the *ipso*-ring.

For the discussed 5,8-dihydroxynaphtho-1,4-quinone **83** and for its tautomeric 4,8-dihydroxynaphtho-1,5-quinone **83T** (Figure 3), a difference between energies of the intramolecular HB (shown in Table S1) of 10.5 kcal/mol and 19.3 kcal/mol, respectively, is observed; however, the last value is distinctive for RAHB systems. Moreover, the quasiHOMA and HOMA indices (0.429 and 0.885, respectively) indicate that while **83** belongs to typical aromatic structures, its tautomer **83T** (with quasiHOMA and HOMA indices at 0.622 and 0.513, respectively), is a particular combination of the two related RAHB couplings. The condensed rings in the tautomer **83T** have no aromatic character and their HOMA indices are uncommonly low. Moreover, on the basis of quasiHOMA values and other hydrogen bonding indicators, e.g., OH∙∙∙O, and O∙∙∙O distances, O-H bond length, spectral and topological properties (see Table S1 and Figures S4–S7), the resonance spacers confirm the non-aromatic character of hydrogen bonding.

It can be suggested that the naphthazarine case, and its enol, are the next example of the transfer of aromaticity from the *ipso*-ring to the external enolic system.

**Figure 4:**
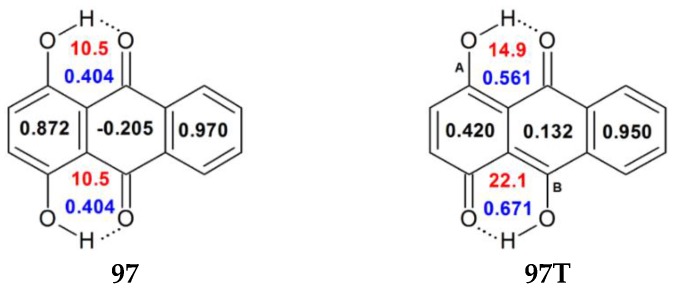
The transfer of aromaticity from *ipso*-ring to the enolic system. Values of quasiHOMA (in blue) and E_HB_ (in red) were given in the centre of the hydrogen bonded ring, whereas the HOMA value was given in the *ipso*-ring.

The hydroxyanthraquinones, known and widely applied as natural pigments and synthetic dyes, are characterized by strong intramolecular hydrogen bonds and values of HOMA indices, which resemble those of quinones (Table 3). Comparison of anthrarufin **96** and quinizarin **97** (Figure 4) indicated that the growth of the quasiHOMA index is accompanied by the decrease of the HOMA for the *ipso*-rings, and that each substituted hydroxyl group increases the strength of intramolecular hydrogen bonding.

Comparison of energies E_HB_, quasiHOMA and HOMA indices of the tautomer of dihydroxyanthraquinone **97T** with those obtained for the tautomer of dihydroxyquinone **83T**, allowed the observation of the same effect of the transfer of aromaticity from two *ipso*-rings. For **97T** HOMA values were reduced to 0.420 and 0.132 (for A and B systems, respectively, to H-bonded rings), due to π-electrons delocalization (quasiHOMA values increased to as much as 0.561 and 0.671 compared to 0.404 in **99** and 0.332 for model salicylaldehyde **1**). The external unsubstituted aromatic ring, not involved in hydrogen bonding, essentially retains its HOMA value.

Many of the analysed structures presented in Table 4 display more than two hydrogen bonds, which were frequently involved in cooperative or chelated systems. In pairs (**104**,**105**), (**107**,**114**), and (**115**,**121**), the additional hydroxyl group of the more substituted molecule “benefits” from the electrons of the same carbonyl oxygen as the first one, which weakens the HB to approximately 1 kcal.

**Figure 5:**
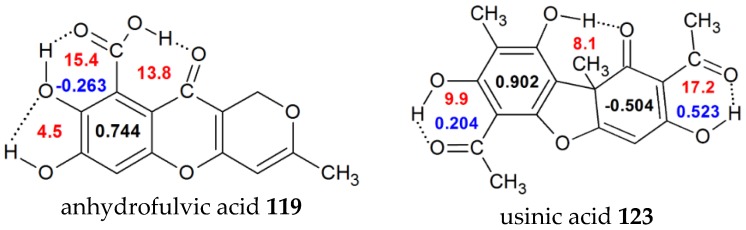
The energies, harmonic oscillator model of aromaticity HOMA and quasiHOMA indices for stable conformers of anhydrofulvic acid **119** (top) and usinic acid **123** (bottom). Values of quasiHOMA (in blue) and E_HB_ (in red) were given in the centre of the hydrogen bonded ring, whereas the HOMA value was given in the *ipso*-ring.

Interestingly, polyfunctional anhydrofulvic acid **119** (Figure 5) is characterized by a planar structure, which can simultaneously form two strong O-H∙∙∙O=C bonds and one O-H∙∙∙O-C_ar_ weak bond in a cooperative hydrogen bonds system. The strongest, with E_HB_ = 15.4 kcal/mol, is not a representative bonding of a phenolic group with carbonyl, due to the fact that it is very short (1.469 Å), it has an extremely long O-H covalent bond (1.017 Å), a significant redshift of ν_OH_ (to 2745 cm^−1^), the highest chemical shift δ_H_ (16.93 ppm), extreme topological parameters at critical points, and is deprived of the quasiaromaticity (quasiHOMA = −0.263) (see Table S1). The reason for such unusual growth of HB energy is the carboxylic group, which forms a seven-membered, extremely short (1.400 Å), and very strong (14.3 kcal/mol) hydrogen bond, with the carbonyl oxygen atom of partially-coupled pyranone. Moreover, both HBs are supported by the five-membered weak O-H∙∙∙O-C_ar_ bond. The unusually long (1.51 Å) spacer (C-C bond) of the carboxylic group with the aromatic ring (1.47 Å for **71** acid) should be noted. In this situation the discussed hydrogen bonding can be better described by topological indices (for example the electron density in RCP, see Supplementary Materials, Table S1) than by the HOMA index, and treated rather as the (−)CAHB related feature [72].

In the study regarding intramolecular hydrogen bonding in naturally-occurring phenolic compounds several structures with seven- or eight-membered rings were also evaluated. Usinic acid **123** (Figure 5) is an example in which three individual hydrogen bonds are isolated: (1) as in *ortho*-hydroxyacetophenone **2** (Table 1) of 9.9 kcal/mol; (2) a typical strong RAHB of 17.2 kcal/mol [25]; and (3) the uncoupled seven-membered H-bonding with E_HB_ of 8.1 kcal/mol equal to nearly half of the RAHB energy.

**Figure 6:**
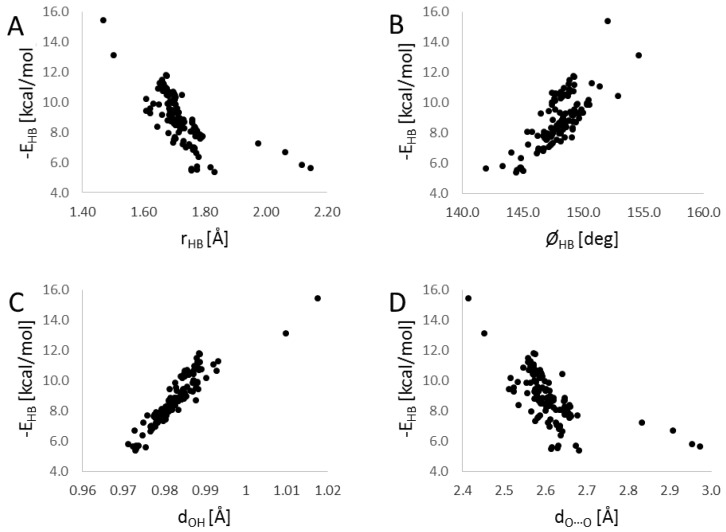

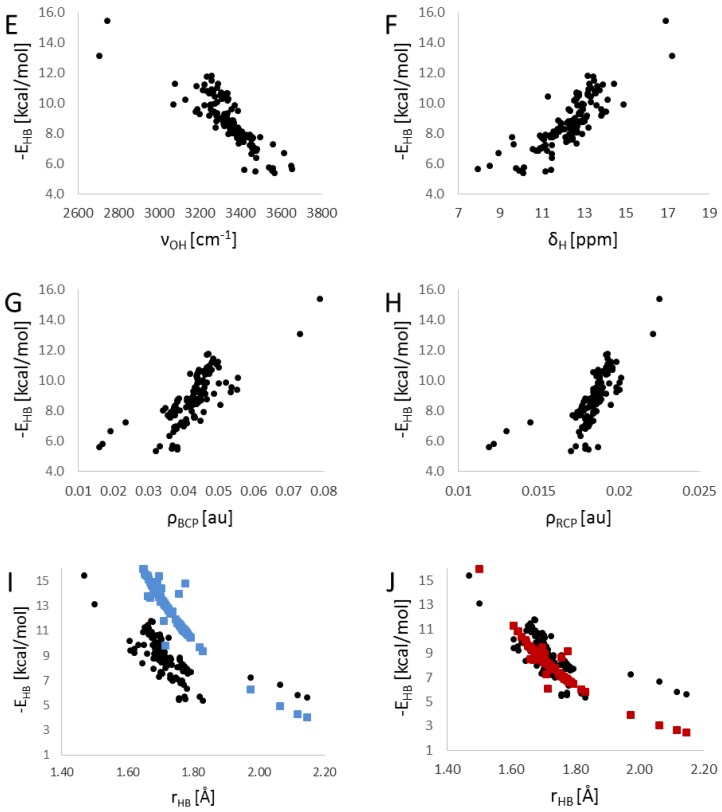
The relations between the calculated energy of intramolecular hydrogen bond E_HB_ (kcal/mol) and some geometrical, spectral and topological parameters of one hundred twenty five investigated hydroxycarbonyl compounds : (**A**) length of H bonding r_HB_ (Å); (**B**) angle of O-H∙∙∙O Ø_HB_ (deg); (**C**) length of O-H bond d_OH_ (Å); (**D**) O∙∙∙O distance d_O∙∙∙O_ (Å); (**E**) frequency of O-H ν_OH_ (cm^-1^); (**F**) chemical shift of O-H hydrogen δ_H_ (ppm), (**G**) electron density in hydrogen bond critical point ρ_BCP_ (au); (**H**) electron density in ring critical point ρ_RCP_ (au); (**I**) comparison of E_HB_ values calculated in the framework of this study and E_HB_ calculated based on Espinosas’ equation ($E_{HB}=\frac{1}{2}V_{BCP}$) [27] in relation to length of H bonding r_HB_ (Å); (**J**) comparison of E_HB_ values calculated in the framework of this study and E_HB_ calculated based on Afonins’ equation ($E_{HB}=0.31\times V_{BCP}$ ) [28] in relation to length of H bonding r_HB_ (Å).

Some of the examined compounds with the phenolic group participating in the seven- or eight-membered rings of intramolecular hydrogen bonding, which are structurally different from the discussed structures, are listed below. They are as follows: 2,3-di(2-hydroxyphenyl)cycloprop-2-en-2-one **124**; 2-(2-hydroxyphenyl)-1,4-benzoquinone **125**; (2-hydroxyphenyl)acetaldehyde **126**; (2-hydroxyphenyl)propanone **127**; *(Z)*-2-hydroxycinnamic acid **128**; and 2,4-bis[4-(*N*,*N*-diethylamino)-2,6-dihydroxyphen-yl]squaraine **129**. Their important parameters are presented at the end of Table S1. They were not excluded from the regression analysis of the completed parameters (Figure 6).

The geometrical and topological parameters designed to describe and measure the strength of intramolecular hydrogen bonding are as follows: the length of O-H∙∙∙O=C bond (r_HB_), the HB angle (*Ø*_HB_), the O∙∙∙O distance (d_O∙∙∙O_), the length of oxygen-hydrogen covalent bond (d_OH_), the frequency of O-H stretching (ν_OH_) and C=O stretching (ν_C=O_), the electron density in the hydrogen bond critical point (ρ_BCP_), its Laplacian (∇^2^ρ_BCP_), the value of potential energy density (V_BCP_), the electron density in the ring critical point (ρ_RCP_), the chemical shift (δ_H_), and the HOMA indices: in the *ipso-* ring (HOMA), and in the hydrogen bonded quasiaromatic ring (quasiHOMA). All of the parameters were collected in Table S1 (Supplementary Materials). Satisfying correlations between hydrogen bond energy E_HB_ and most of the listed parameters were found, and the best matches are presented in Figure 6.

It can be seen, in Figure 6, that there is a relatively good correlation for the majority of the studied structures, with the exception of **119** and **123** (discussed above), which are characterized by the strongest H bonding, as well as structures with a weak H bonding: 7-hydroxy-1-indanone **62**, salfredin **103**, 1-hydroxyfluorenone **105**, and 1,8-dihydroxyfluorenone **104**. The last four compounds have the H-bonding systems stiffened by external five-membered rings, therefore, they do not match the main trend due to the geometric constraints of HB (see Supplementary Materials, Figure S1, structure **62**).

The best correlations were established between the energy of the intramolecular H bond and the length of the covalent O-H bond, as well as the frequency of O-H stretching. These parameters may be safely employed for estimation of the strength of HB. The NMR chemical shift values (empirical or calculated) of the analysed structures, which are often successfully used to compare the strength of HB [28,73], were mostly similar with the results obtained in our study (Supplementary Materials, Table S1) and may be correlated with the rest of typical parameters used in HB examination. The values of HOMA were within a range of 0.504 to 0.978, whereas quasiHOMA ranged from −0.236 to 0.543, but they were not sufficiently correlated with E_HB_ (see Supplementary Materials, Figure S2).

## 4. Summary and Discussion

The problem associated with quantitative measurements of the strength of intramolecular hydrogen bonding energy is widely recognized. However, it has not been definitively resolved to date. Our previous studies were focused on an attempt to take advantage of the computational method of the molecular tailoring approach (MTA), which has been previously proposed and applied for hydrogen bonds in hydroxy–hydroxy or hydroxy–alkoxy systems by [18]. This method is based on quantum chemistry calculations of the energy of molecules as a sum of their smaller fragments.

When the molecule is entangled in the intramolecular hydrogen bond and “this internal HB can be broken without causing other structural or electronic changes” [11], as in the MTA method, the calculations are possible for each combination of O-H as a donor, and O=C as an acceptor, of HB. When the donor, the acceptor, and both are replaced by the hydrogen atom in the same molecule, their energies may be summarized and compared with the energy calculated for the whole molecule [30]. The conditions which are necessary for the success of this method include preliminary optimization of the molecule, appropriate incision of the system, and no intervention in geometry after insertion of hydrogen atoms. This method has been previously checked for many hydrogen bonds present in hydroxycarbonyl compounds with the full possibility of rotation (saturated) or with some restraints (double bond, ring) [30] and recently, for several structures with resonance-assisted hydrogen bond (RAHB) [25], good accuracy, repeatability, and applicability have been found.

As mentioned in the paragraph following Table 1, the E_HB_ values presented in the literature by different methods are divergent. The most widely used method (*cis*-*trans*, *syn*-*anti*, open-close) is based on the comparison of energies of two conformers: one involved in the intramolecular HB, and the second one in which this bond is broken by the rotation of the proton donor OH group outside of the carbonyl acceptor. This method is flawed because it does not include the repulsion of both oxygen atoms. It cannot be used for more complex compounds with more than one HB and with the steric hindrance of the rotated OH group. The results are mostly overestimated, in some cases even up to two times [16,44,54,59].

When experimental or calculated parameters (length of hydrogen bonds, the O-H covalent bond length, the O∙∙∙O distance, IR frequencies, NMR chemical shifts) have been included in the estimation [56,62,63,69,73], the E_HB*cis-trans*_ values are smaller and similar to those obtained in this study. Although the isodesmic approach results in values similar to those obtained in the framework of this study, their use for estimating the HB energy in such complicated structures is not recommended. This method is advocated for systems with one HB, but is not recommended for the estimation of the single intramolecular H-bond energy in polyhydroxy systems [17]. Values of interaction energies obtained by means of isodesmic reactions seem to be more reliable if they concern planar molecules [18]. Over the last two decades, the methods based on the AIM electron density parameters, which may be measured for covalent bonds, for hydrogen bonds, and for aromatic rings, have been considered as advantageous. The EHB energy can also be calculated on the basis of the potential energy density V_BCP_ by Espinosa’s [27] equation, which was recently examined in detail and modified by Afonin [28] using new linear regression coefficients.

Energies of intramolecular H-bonds presented in this study comply well with the values calculated based on Espinoza’s equation modified by Afonin ($E_{HB}=0.31\times V_{BCP}$) [28] (see Figure 6J).

In the presented work, the MTA method was used to analyse the hydrogen bonding and to calculate HB energy for phenols (hydroxyphenyl group as a proton donor) and miscellaneous carbonyl spacers. Almost 140 structures of hydroxyaromatic aldehydes, ketones, acids, esters, amides, quinones, and anthraquinones were optimized at the MP2(FC)/6-311++G(2d,2p) level, then cut into appropriate fragments and E_HB_ calculated with the MTA method. The results obtained for simple phenolic, substituted, and more elaborate structures can be arranged in a sequence congruent with geometrical, spectral, and topological indicators of the hydrogen bonding strength. The MTA energies of the intramolecular hydrogen bond between phenolic hydrogen and carbonyl groups in the six-membered ring are in the range of 5.4 to 15.4 kcal/mol. When the phenolic group participates as a donor in seven- or eight-membered HB rings, the E_HB_ typically ranges from 4.6 to 9.6 kcal/mol, and does not differ from values obtained for saturated carbonyl substituted alcohols.

For all H-bonded phenolic structures, the HOMA indices of aromaticity were calculated for the *ipso*-rings [12], while quasiHOMA indices were calculated for the H-bonded rings. Changes of both parameters associated with substitution in the *ipso*-ring and elsewhere, as well as the effects of cooperation and/or chelation of HB were observed, and it was noticed that quasiHOMA varied from negative values to 0.543, whereas HOMA ranged from 0.503 to 0.978, but they were not correlated with E_HB_. This is not surprising, because this parameter was calculated according to Equation (2) on the basis of the length of four bonds: C_ar_-O, C_ar_-C_ar_, C_ar_-C(=O), and C=O, and does not specify the position of the H-bonded hydrogen atom. In some of the analyzed structures important regularity was found. The presented pairs of tautomers of triformylphloroglucinol **44** and 2,4,6-tri(hydroxymethylindene)cyclohexa-1,3,5-trione **44T**, as well as triacetylophloroglucine **70** and triacetophen **70T**, evidently showed that total resonance stabilization energy is defined and limited by the heavy atom frame of the molecule. The transfer of phenolic protons (within the frame) leads to the formation of a related tautomer which differs—by definition—in terms of the localization of acidic (i.e., phenolic or enolic) protons and, consequently, the pattern of carbon-carbon double bonds. The tautomerization of aromatic derivative due to *inter alia* π-electron transfer generally canceled the aromaticity of the central ring, but its resonance energy transfers outside this ring—into its rearranged, but still H-bonded substituents—organized into three external rings.

Similarly, the analysis of hydrogen bonding of 1,4-benzoquinone derivatives and their tautomers (pairs **83** and **83T**, and **97** and **97T**) led to a similar picture of the redistribution of π-delocalization from benzene into HB rings (Figure 3 and Figure 5) which was manifested by the decrease of HOMA indices and a notable increase of quasiHOMA and E_HB_ values. The phenomenon of transfer of aromaticity can be rationalized in terms of the number of π-electrons counted for each ring: in Figure 4 structure **97** of quinizarin has two benzenoid rings with six π-electrons each, thus, being fully aromatic according to the Hückel rule, and a central ring with only four π-electron is not aromatic. It is, thus, not unexpected that the HOMA indices of the first are 0.872 and 0.970, while for the last a value of −0.205 is found. On passing for the structure **97T** a ring with six π-electrons is maintained, with a HOMA value of 0.950, but now the other benzenoid ring has only five π-electrons and its HOMA is, accordingly, 0.420. On this hand the central ring, also with five π-electrons, has a quasiHOMA value of 0.132.

The calculation of HOMA and quasiHOMA indicated that spectacular losses of resonance energy in the aromatic ring was found as a result of the large increase of concomitant quasiaromaticity of the two rearranged hydrogen bonding systems present. In other words, the E_HB_ values typical for very strong RAHB hydrogen bonds and appreciable quasiHOMA values confirmed the origin of the reduced HOMA values of the enolic tautomer.

Finally, this study shows that introduction of the energy of hydrogen bonds calculated based on the MTA method as the parameter characterizing the molecular system reveals new possibilities for the interpretation of the associated resonance-assisted phenomena.

## Figures and Tables

**Table 1:** Harmonic Oscillator Model of Aromatic stabilization (HOMA) and quasiHOMA indices and energy of the hydrogen bond (kcal/mol), calculated for unsubstituted *ortho*-hydroxycarbonyl compounds, substituted *ortho*-hydroxyaldehydes, substituted hydroxyketones and acids.

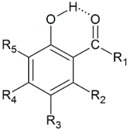									
Unsubstituted *o*-Hydroxycarbonyl Compounds									
No	R_1_	HOMA	quasiHOMA	E_HB_^a^	Ref.				
**1**	H	0.953	0.332	7.9	8.37 [53], 10.61 [54], 9.62 [55], 12.66 [56],11.08 [40],10.9 [37], 11.07 [39], 5.64 [57], 12.33 [44], 10.33 [58], 15.60 [59], 7.1 [28]				
**2**	Me	0.940	0.180	8.6	16.4 [34], 14.23 [44], 10.55 [54] ,9.5 [28]				
**3**	Ph	0.937	0.187	8.2	6.52, 5.64, 5.47 [60]				
**4**	OH	0.953	0.211	6.8	10.95 [61], 12.17 [44], 11.66 [58], 5.8 [62]				
**5**	OMe	0.954	0.160	7.1	12.28 [44], 6.8 [63], 7.1 [28]				
**6**	OEt	0.954	0.151	7.2					
**7**	OPh	0.952	0.174	7.0	6.4 [62]				
**8**	NH_2_	0.950	0.090	8.6	15.42 [44]				
**9**	NHMe	0.956	0.047	8.1	16.0 [44]				
**10**	NHPh	0.949	0.035	8.0					
**11**	N(Me)_2_	0.960	0.001	7.8					
**12**	SH	0.947	0.200	7.0	11.34 [48], 11.96 [44]				
**13**	SMe	0.946	0.175	7.2	12.39 [44]				
**14**	F	0.952	0.204	5.7	10.13 [44]				
**15**	Cl	0.937	0.189	5.7	9.64 [44]				
**16**	Br	0.934	0.176	5.5					
**17**	CN	0.935	0.369	6.6	10.97 [44]				
**18**	NO_2_	0.942	0.347	5.4	9.68 [44]				
**Substituted *o*-Hydroxyaldehydes**									
**No**	**R_1_**	**R_2_**	**R_3_**	**R_4_**	**R_5_**	**HOMA**	**quasiHOMA**	**E_HB_^a^**	**Ref.**
**19**	H	H	Me	H	H	0.948	0.339	7.9	15.00 [59]
**20**	H	H	OHtrans	H	H	0.956	0.300	7.6	8.17 [53], 14.37 [59]
**21**	H	H	OHcis	H	H	0.952	0.332	7.9	8.69 [53]
**22**	H	H	NH_2_	H	H	0.948	0.321	7.7	14.15 [59]
**23**	H	H	N(Me)_2_	H	H	0.929	0.323	7.7	14.85 [59]
**24**	H	H	F	H	H	0.954	0.310	7.8	9.650 [55], 14.84 [59]
**25**	H	H	Cl	H	H	0.959	0.314	7.8	15.23 [59]
**26**	H	H	Br	H	H	0.959	0.402	7.8	15.31 [59]
**27**	H	H	C(O)H	H	H	0.951	0.294	7.7	
**28**	H	H	H	C(O)H	H	0.942	0.321	7.9	
**29**	H	H	NO_2_	H	H	0.960	0.302	8.0	8.28 [53], 16.48 [59]
**30**	H	H	H	Me	H	0.948	0.352	8.1	10.679 [54]
**31**	H	H	H	OH	H	0.953	0.399	8.7	8.55 [53], 10.867 [54]
**32**	H	H	H	OMe	H	0.948	0.371	8.1	
**33**	H	H	H	NH_2_	H	0.938	0.412	8.7	11.276 [54]
**34**	H	H	H	N(Me)_2_	H	0.897	0.427	8.8	11.13 [54]
**35**	H	H	H	F	H	0.958	0.365	8.3	9.93 [55], 10.895 [54]
**36**	H	H	H	Cl	H	0.957	0.359	8.1	10.722 [54]
**37**	H	H	H	Br	H	0.957	0.357	8.0	10.685 [54]
**38**	H	H	H	NO_2_	H	0.953	0.330	7.9	7.91 [53], 10.393 [54]
**39**	H	H	F	H	F	0.953	0.334	7.8	6.38 [55]
**40**	H	F	H	F	H	0.950	0.369	8.9	10.47 [55]
**41**	H	OH	H	OH		0.944	0.418	9.4	
**42**	H	OH trans	H	H	H	0.945	0.339	8.6	12.57 [48]
**43**	H	-O-(CO)-CH=CH-		H	H	0.929	0.339	8.9	
**44**	H	OH	C(O)H	OH	C(O)H	0.884	0.494	11.3	triformylphloroglucinol
**45**	H	H	H	NH_3_^+^	H	0.954	0.224	8.1	
**46**	H	H	NH_3_^+^	H	H	0.946	0.257	8.1	16.88 [59], 10.7 [54]
**47**	H	H	H	O^-^	H	0.504	0.081	10.5	
**48**	H	H	O^-^	H	H	0.696	0.263	7.7	12.36 [59], 12.05 [54]
**44T**	H	enolic tautomer of **44** ^b^				-0.022	0.631	18.3	
**Substituted Hydroxyketones and Acids**									
**No**	**R_1_**	**R_2_**	**R_3_**	**R_4_**	**R_5_**	**HOMA**	**quasiHOMA**	**E_HB_^a^**	**Ref. and/or Common Name**
**49**	Me	OHcis	H	H	H	0.881	0.128	8.4	
**50**	Me	OHtrans	H	H	H	0.906	0.086	9.3	
**51**	Me	OMetrans	H	H	H	0.887	0.099	9.4	
**52**	Me	OH	H	Me	H	0.909	0.130	9.6	
**53**	Me	H	H	OHtrans	H	0.942	0.267	9.4	
**54**	Me	H	H	OMecis	H	0.938	0.234	8.8	
**55**	Me	H	H	-CH_2_CH=CHCH_2_-		0.928	0.215	9.2	
**56**	Ph	H	H	OMecis	H	0.933	0.250	8.5	7.19, 7.8, 8.5 [64]
**57**	Ph	H	Cl	Me	H	0.942	0.194	8.3	
**58**	CH=CH-Ph	H	H	H	H	0.927	0.164	9.2	
**59**	CH=CH-Ph	OMe	H	OMe	H	0.891	0.256	9.9	chalcone
**60**	CH=C(OH)Ph	H	H	H	H	0.931	0.184	8.0	
**61**	(*o*-OH)Ph	H	H	H	H	0.939	0.176	6.4	
**62**	-CH_2_-CH_2_-		H	H	H	0.978	0.314	7.2	
**63**	-CH_2_-CH(OH)-CH_2_‑		H	H	H	0.929	0.220	8.8	vermelone ^c^
**64**	-CH=CH-O-		H	H	H	0.948	0.328	9.0	
**65**	-CH_2_-CH(OH)-CH_2_-		H	OH	H	0.935	0.345	9.4	scytalone ^c^
**66**	-CH_2_-CH(OH)-CH_2_-		H	OH	H	0.932	0.312	9.6	scytalone ^c^
**67**	-CH_2_-CH_2_-C=O		OH	H	H	0.875	0.327	9.6	
**68**	-(CH=CH)-C=CH_2_		OH	H	H	0.920	0.239	8.8	
**69**	Me	OH	H	OH	H	0.906	0.192		acetylphloroglucinol
**70**	Me	OH	C(O)Me	OH	C(O)Me	0.774	0.303	13.1	triacetylphloroglucinol
**71**	OH	H	F	H	H	0.955	0.196	7.0	10.89 [61]
**72**	OH	H	Cl	H	H	0.959	0.197	6.9	10.76 [61]
**73**	OH	H	Br	H	H	0.959	0.198	6.9	10.94 [61]
**70T**	enolic tautomer of **70** ^d^					−0.044	0.635	21.9	

^a^ all E_HB_ were given as positive values; ^b^ described in [25] as compound 170; ^c^ scytalone isomers OH 60deg and 175deg; vermelone [35]; ^d^ described in [25] as compound 171.

**Table 2:** HOMA and quasiHOMA indices and energy of hydrogen bond (kcal/mol) calculated for hydrogen bonding (HB) ring in substituted hydroxynaphthoquinones structures; common names of some natural products and the cited energetic data were given in the last column.

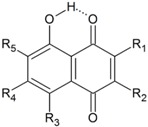									
No	R_1_	R_2_	R_3_	R_4_	R_5_	HOMA	quasiHOMA	E_HB_	Common Name and Ref.
**74**	H	H	H	H	H	0.938	0.313	8.3	juglone
**75**	H	CH_3_	H	H	H	0.943	0.317	8.7	plumbagin
**76**	H	(CH_2_)_3_COOH	H	H	H	0.942	0.322	8.5	
**77**	H	NH(CH_2_)_2_COOH	H	H	H	0.946	0.207	9.9 ^a^	juglonbutin
**78a**	CH_2_-COOH	OH	H	H	H	0.944	0.290	8.5 ^b^	grecoketide
**78b**	CH_2_-COOH	OH	H	H	H	0.945	0.283	8.5 ^c^	grecoketide
**78c**	CH_2_-COOH	OH	H	H	H	0.936	0.253	8.8 ^d^	grecoketide
**79**	CH_2_CH=C(CH_3_)_2_	OH	H	H	H	0.943	0.226	9.3 ^e^	hydroxylapachol
**80**	CH_2_CH=C(CH_3_)_2_	OH	H	H	H	0.945	0.260	8.7 ^f^	hydroxylapachol
**81**	H	(1,3-diOH)Ph	H	H	H	0.946	0.330	8.9	juglone
**82**	-O-C(CH_3_)_2_-(CH_2_)_2_-		H	H	H	0.942	0.325	8.4	caryopterone
**83**	H	H	OH	H	H	0.885	0.429	10.5	naphthazarin 13.0 [37], 19.36 [68], 13.15 [69], 14.5 [70]
**84**	CH_2_-CO-CH_3_	CH_3_	OH	H	OCH_3_	0.868	0.524	11.7 ^g^	javanicin
						0.868	0.543	11.8	
**85**	Cl	H	OH	H	H	0.883	0.437	10.3 ^g^	13.5, 14.0 [70]
						0.883	0.450	10.7	
**86**	H	H	OH	Cl	H	0.883	0.450	10.3 ^g^	13.9, 11.2 [70]
						0.883	0.441	10.7	
**87**	Cl	Cl	OH	H	H	0.884	0.466	10.6	12.8 [70]
**88**	H	H	OH	Cl	Cl	0.823	0.453	10.6	11.8 [70]
**89**	Cl	H	OH	H	Cl	0.881	0.451	10.4 ^g^	10.6, 13.8 [70]
						0.881	0.474	10.6	
**90**	H	Cl	OH	H	Cl	0.880	0.461	10.9 ^g^	13.3, 11.2 [70]
						0.880	0.464	10.2	
**91**	Cl	Cl	OH	Cl	H	0.880	0.489	10.6 ^g^	10.7, 13.4 [70]
						0.880	0.481	11.0	
**92**	Cl	H	OH	Cl	Cl	0.874	0.490	10.5 ^g^	10.6, 11.3 [70]
						0.874	0.497	10.8	
**93**	Cl	Cl	OH	Cl	Cl	0.874	0.513	10.7	11.2 [70]
**83T**	4,8-dihydroxynaphtho-1,5-quinone					0.513	0.622	19.32	

^a^ additional E_HB_ NH to =O4 5-membered 12.6 kcal/mol and NH to carboxylic =O 6-membered 0.5 kcal/mol; ^b^ additional E_HB_ R_2_(O-H) to R_1_(OH) 7-membered 3.9 kcal/mol; ^c^ additional E_HB_ R_2_(O-H) to R_1_(C=O) 7-membered 6.7 kcal/mol; ^d^ additional E_HB_ R_1_ (carboxylic OH) to R_2_ 8-membered 4.7 kcal/mol; ^e^ additional E_HB_ R_2_(OH) to O4 5-membered 8.41; ^f^ additional E_HB_ R_2_(OH) to C=C in R_1_ substituent 4.5 kcal/mol; ^g^ two HB, not identical because of Cl position.

**Table 3:** HOMA and quasiHOMA indices and energy of hydrogen bond (kcal/mol) calculated for HB rings in substituted hydroxyanthraquinones.

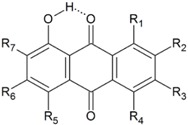										
No	R_1_	R_2_	R_3_	R_4_	R_5_	R_6_	R_7_	HOMA	quasiHOMA	E_HB_
**94**	H	H	H	H	H	H	H	0.929	0.301	8.8 ^a^
**95**	H	H	H	H	H	H	OH	0.939	0.310	10.0
**96**	H	H	H	OH	H	H	H	0.933	0.316	9.1 ^b^
**97**	H	H	H	H	OH	H	H	0.872	0.404	10.5 ^c^
**98**	OH	H	H	H	H	H	H	0.927	0.344	7.6
**99**	OH	H	H	OH	OH	H	H	0.870	0.448	9.9
**100**	OH	OH	H	H	OH	H	H	0.740	0.366	9.3 ^d^
								0.870	0.458	9.5 ^d^
								0.870	0.388	11.5 ^d^
**101**	H	OH	COOH	CH_3_	OH	H	OH	0.871	0.518	11.6 ^e^
								0.871	0.448	10.9 ^e^
								0.881	0.024	5.6 ^e^
**97T**	H	H	H	H	R_5_=O, R_10_=OH	H	H	0.420	0.561	14.9
								0.132	0.671	22.1

^a^ E_HB_ = 13.36 kcal/mol in [58]; ^b^ anthrarufin; ^c^ quinizarin; ^d^ quinalizarin; three HB: two chelated to O9 and one to O10, add. 5-membered ring R_2_(O-H) to R_1_(O-H) 2.6 kcal/mol in cooperation; ^e^ carmin acid without glicoside; two HB to O9 and O10; add. R_2_(O-H) to R_3_(C=O) 6-membered ring 5.6 kcal/mol.

**Table 4:** Energy of hydrogen bond (kcal/mol) as well as HOMA and quasiHOMA indices calculated for HB rings for some biological active phenols.

No	Name (Customary Name)	HOMA	quasiHOMA	E_HB_	Ref.
**102**	2-hydroxy-6-methyl [(1*R*)-2-oxo-(5*R*,6*R*)-5,6-dihydroxy-3-methyl)cyclohex-3-enoic]benzoate	0.914	0.163	5.5 ^che^	
**103**	5-hydroxy-2,2-dimethyl-8*H*-furo[3,4-g]chromen-6-one (salfredin B)	0.990	0.205	5.6	
**104**	1,8-dihydroxyfluoren-9-one	0.954	0.218	5.8 ^che^	
**105**	1-hydroxyfluoren-9-one	0.960	0.169	6.7	
**106**	1,8-dihydroxy-9,10-dihydroanthracen-9-one (dithranol)	0.930	0.286	7.3 ^che^	
**107**	1,8-dihydroxy-9*H*-xanthen-9-one	0.939	0.422	7.4 ^che^	
**108**	8-hydroxy-2-(1-hydroxyethylene)-3,6-dimethyl-1(2*H*)-naphthalenone	0.900	0.412	7.6 ^che^	9.5 [71]
**109**	8-hydroxy-2-(1-hydroxyethylene)-1(2*H*)-naphthalenone	0.885	0.397	7.7 ^che^	
**110**	2,3-dihydro-2,5-dihydroxy-4*H*-benzopyran-4-one (DDBO in [35])	0.939	0.310	8.2	
**111**	3,5,7-trihydroxy-2-(4-hydroxyphenyl)-3,5,7-trihydroxy-4*H*-chromen-4-one (kemferol)	0.943	0.529	8.3 ^che^	
**112**	2-(3,4-dihydroxyphenyl)-3,5,7-trihydroxy-4*H*-chromen-4-one (quercetin)	0.944	0.528	8.4	
**113**	(1S*,3aR*,9aS*)-1,8-dihydroxy-1,2,3,3a-tetrahydrocyclopenta-[b]chromen-9(9a*H*)-one (diaporteone B)	0.937	0.385	8.7 ^che^	
**114**	1-hydroxy-9*H*-xanthen-9-one	0.788	0.366	8.7	
**115**	9(10*H*)-1,8-dihydroxyacridinone	0.921	0.452	8.8 ^che^	4.97 [23]
**116**	7-hydroxy-2,2-dimethyl-3,4-dihydro-2*H*-benzo[h]chromene-5,6-dione (7-hydroxylapachone)	0.933	0.363	9.3	
**117**	5-hydroxy-4*H*-chromen-4-one	0.952	0.347	9.3	
**118**	2-(3,4-dihydroxyphenyl)-5,7-dihydroxy-4-chromenone (luteolin)	0.944	0.398	9.8	
**119**	7,8-dihydroxy-3-methyl-10-oxo-1*H*,10*H*-pyrano[4,3-b]chromene-9-carboxylic acid (anhydrofulvicacid)	0.744	-0.263	15.4	
**120**	5,7-dihydroxy-3-(4-hydroxyphenyl)chromen-4-one (genistein)	0.941	0.392	10.0	
**121**	5-hydroxy-4-quinolon	0.921	0.299	11.1	5.7 [23]
**122**	2,3-dihydro-9,10-dihydroxy-1,4-anthracenedione (leucoquinizarin)	0.692	0.434	11.2	
**123**	2,6-diacetyl-7,9-dihydroxy-8,9b-dimethyl-1,3(2*H*,9bh) (usinicacid)	0.902	0.204	9.9	20.1 [34]

^che^ indicates a carbonyl chelated hydrogen bonding.

## Supplementary Materials

The following are available online at www.mdpi.com/1420-3049/22/3/481/s1, Figure S1: Examples of MTA fragmentation employed for selected structures analyzed in the study. Different colors represent different fragmentation of different intramolecular hydrogen bonds, Figure S2: MTA Intramolecular Hydrogen Bond Energy [kcal/mol] as a Function of Laplacian of the Electron Density in the Bond Critical Point [au] (A) and its HOMA Index (B) for Structures with Phenolic Intramolecular Hydrogen Bonding, Table S1: MTA Energy of Intramolecular Hydrogen Bonding EHB [kcal/mol], Length of the HB as H···O (r_HB_) [A], Angle of the HB as O-H···O (φ_HB_)[deg], Length of the O-H Bond (d_OH_) [A], Distance Between the Oxygen Atoms as O···O (d_O__···__O_) [A], Frequency of O-H and C=O Stretching [cm^-1^], HNMR Chemical Shifts δ_H_ [ppm], Electron Density in the Bond Critical Point (ρ_BCP_) [au] and its Laplacian (∇^2^ρ_BCP_), the value of Potential Energy Density (V_BCP_), Electron Density in the Ring Critical Point (ρ_RCP_) [au] and HOMA and quasiHOMA Indices Calculated for Structures **1** – **129**. Structures **44T**, **70T**, **83T** and **97T** serve to compare them with **44**, **70**, **83**, **97** because they not present phenolic type of HB.
