# Supplementary material for: Energy of Intramolecular Hydrogen Bonding in ortho-Hydroxybenzaldehydes, Phenones and Quinones. Transfer of Aromaticity from ipso-Benzene Ring to the Enol System(s)
Source: Molecules. 2017 Mar 18;22(3):481. doi: 10.3390/molecules22030481 (PMC6155192; doi:10.3390/molecules22030481)
Supplement: Supplementary file 1 [file molecules-22-00481-s001.pdf]

Supplementary material for

Energy of Intramolecular Hydrogen Bonding in ortho-Hydroxybenzaldehydes, Phenones and Quinones. Transfer of Aromaticity from ipso-Benzene Ring to the Enol System(s).

Danuta Rusinska-Roszak

Institute of Chemical Technology and Engineering, Poznan University of Technology, Ul. Berdychowo 4,

60-965 Poznan, Poland

* Corresponding author: E-mail: danuta.rusinska-roszak@put.poznan.pl
Table of Contents

Table of Contents

Table S1. MTA Energy of Intramolecular Hydrogen Bonding EHB [kcal/mol], Length of the HB as H···O (r_{HB}) [A], Angle of the HB as O-H···O (φ_{HB})[deg], Length of the O-H Bond (d_{OH}) [A], Distance Between the Oxygen Atoms as O···O ($d_{O\cdots O}$) [A], Frequency of O-H and C=O Stretching [cm^{-1}], HNMR Chemical Shifts (δ_H) [ppm], Electron Density in the Bond Critical Point (ρ_{BCP}) [au] and its Laplacian ($\nabla^2\rho_{BCP}$), the value of Potential Energy Density (V_{BCP}), Electron Density in the Ring Critical Point (ρ_{RCP}) [au] and HOMA and quasiHOMA Indices Calculated for Structures **1 – 129.** Structures **44T, 70T, 83T** and **97T** serve to compare them with **44, 70, 83, 97** because they not present phenolic type of HB.

Figure S1. Examples of MTA fragmentation employed for selected structures analyzed in the study. Different colors represent different fragmentation of different intramolecular hydrogen bonds.

Figure S2. MTA Intramolecular Hydrogen Bond Energy [kcal/mol] as a Function of Laplacian of the Electron Density in the Bond Critical Point [au] (A) and its HOMA Index (B) for Structures with Phenolic Intramolecular Hydrogen Bonding.

Table S1. MTA Energy of Intramolecular Hydrogen Bonding EHB [kcal/mol], Length of the HB as H···O (r_{HB}) [A], Angle of the HB as O-H···O (φ_{HB})[deg], Length of the O-H Bond (d_{OH}) [A], Distance Between the Oxygen Atoms as O···O ($d_{O\cdots O}$) [A], Frequency of O-H and C=O Stretching [cm^{-1}], HNMR Chemical Shifts (δ_H) [ppm], Electron Density in the Bond Critical Point (ρ_{BCP}) [au] and its Laplacian ($\nabla^2\rho_{BCP}$), the value of Potential Energy Density (V_{BCP}), Electron Density in the Ring Critical Point (ρ_{RCP}) [au] and HOMA and quasiHOMA Indices Calculated for Structures **1 – 129.** Structures **44T, 70T, 83T** and **97T** serve to compare them with **44, 70, 83, 97** because they not present phenolic type of HB.

no	$-E_{HB}$	r_{HB}	$Ø_{HB}$	d_{OH}	ν_{OH}	$\nu_{C=O}$	$d_{O\cdots O}$	ρ_{BCP}	$\nabla^2\rho_{BCP}$	V_{BCP}	ρ_{RCP}	δ_H	HOMA	quasi HOMA
1	**7.85**	1.7724	147.43	0.9791	3427	1706	2.650	0.0372	0.1328	3.523	0.0176	11.617	0.9531	0.3316
2	**8.57**	1.7003	148.25	0.9821	3333	1684	2.588	0.0443	0.1513	4.364	0.0186	12.654	0.9395	0.1796
3	**8.20**	1.7207	147.56	0.9806	3352	1664	2.602	0.0349	0.1551	4.103	0.0180	12.655	0.9367	0.1868
4	**6.82**	1.7610	146.67	0.9770	3474	1728	2.633	0.0380	0.1381	3.632	0.0179	11.244	0.9533	0.2110
5	**7.09**	1.7516	147.15	0.9779	3462	1709	2.627	0.0390	0.1403	3.741	0.0179	11.076	0.9543	0.1603
6	**7.23**	1.7474	147.35	0.9781	3458	1705	2.625	0.0394	0.1411	3.788	0.0180	11.155	0.9545	0.1514
7	**6.99**	1.7637	146.67	0.9769	3471	1723	2.635	0.0379	0.1378	3.623	0.0180	10.913	0.9524	0.1738
8	**8.63**	1.6980	148.55	0.9829	3317	1699	2.588	0.0443	0.1509	4.361	0.0188	12.456	0.9496	0.0897
9	**8.06**	1.7610	146.78	0.9794	3362	1684	2.635	0.0382	0.1355	3.630	0.0185	11.678	0.9563	0.0467
10	**7.99**	1.7157	147.99	0.9812	3357	1689	2.600	0.0424	0.1475	3.131	0.0186	12.453	0.9492	0.0347
11	**7.83**	1.7839	146.25	0.9783	3428	1668	2.654	0.0363	0.1303	3.408	0.0184	10.910	0.9596	-0.0008
12	**6.98**	1.7375	146.76	0.9778	3459	1679	2.611	0.0402	0.1440	4.002	0.0181	10.990	0.9466	0.1999
13	**7.20**	1.7289	147.29	0.9785	3446	1670	2.606	0.0411	0.1458	4.002	0.0182	11.470	0.9456	0.1747
14	**5.69**	1.8195	144.76	0.9732	3560	1795	2.674	0.0333	0.1260	3.089	0.0173	9.778	0.9521	0.2040
15	**5.72**	1.7740	144.84	0.9738	3542	1751	2.631	0.0369	0.1376	3.523	0.0179	10.140	0.9372	0.1886
16	**5.53**	1.7755	144.49	0.9732	3558	1757	2.629	0.0367	0.1345	3.508	0.0179	9.910	0.9343	0.1763
17	**6.64**	1.7720	146.15	0.9767	3456	1673	2.640	0.0371	0.1260	3.548	0.0175	11.493	0.9351	0.3694
18	**5.38**	1.8303	144.44	0.9729	3568	1768	2.682	0.0322	0.1242	2.992	0.0170	10.106	0.9418	0.3467
19	**7.88**	1.7781	147.23	0.9788	3426	1704	2.654	0.0368	0.1318	3.468	0.0175	11.539	0.9479	0.3389
20	**7.56**	1.7840	146.96	0.9778	3453	1709	2.658	0.0362	0.1311	3.412	0.0173	11.087	0.9555	0.3003
21	**7.92**	1.7808	147.03	0.9781	3446	1709	2.655	0.0365	0.1317	3.445	0.0174	11.178	0.9522	0.3318
22	**7.74**	1.7865	146.96	0.9777	3457	1707	2.660	0.0360	0.1305	3.390	0.0173	11.000	0.9479	0.3212
23	**7.67**	1.7898	146.85	0.9775	3464	1706	2.662	0.0358	0.1298	3.360	0.0173	11.016	0.9289	0.3235
24	**7.84**	1.7769	147.04	0.9784	3438	1712	2.652	0.0368	0.1324	3.480	0.0175	11.380	0.9544	0.3105
25	**7.75**	1.7763	147.04	0.9788	3429	1711	2.652	0.0369	0.1323	3.483	0.0175	11.399	0.9586	0.3139
26	**7.75**	1.7757	147.08	0.9789	3429	1710	2.651	0.0370	0.1323	3.488	0.0175	11.527	0.9589	0.4023
27	**7.74**	1.7683	147.37	0.9800	3387	1710	2.647	0.0376	0.1333	3.558	0.0176	12.114	0.9512	0.2939
28	**7.87**	1.7709	147.05	0.9795	3419	1710	2.647	0.0374	0.1334	3.538	0.0176	11.674	0.9417	0.3210
29	**7.97**	1.7713	147.01	0.9799	3375	1714	2.648	0.0373	0.1326	3.526	0.0176	12.300	0.9603	0.3022
30	**8.11**	1.7746	147.52	0.9794	3406	1699	2.653	0.0371	0.1320	3.501	0.0176	11.620	0.9483	0.3523
31	**8.65**	1.7597	148.04	0.9811	3358	1693	2.644	0.0384	0.1343	3.643	0.0178	11.933	0.9531	0.3985
32	**8.09**	1.7690	148.06	0.9800	3400	1696	2.652	0.0376	0.1327	3.550	0.0177	11.552	0.9477	0.3715
33	**8.71**	1.7601	148.35	0.9811	3349	1685	2.646	0.0384	0.1341	3.640	0.0179	11.881	0.9382	0.4116
34	**8.83**	1.7579	148.81	0.9816	3358	1681	2.646	0.0387	0.1341	3.667	0.0179	11.774	0.8975	0.4266
35	**8.28**	1.7648	147.67	0.9804	3366	1703	2.646	0.0379	0.1339	3.593	0.0177	11.963	0.9580	0.3654
36	**8.11**	1.7647	147.57	0.9803	3400	1704	2.645	0.0379	0.1342	3.596	0.0177	11.837	0.9574	0.3586
37	**7.99**	1.7662	147.52	0.9802	3403	1704	2.646	0.0378	0.1340	3.580	0.0177	11.680	0.9573	0.3567
38	**7.87**	1.7714	147.03	0.9796	3432	1717	2.648	0.0373	0.1335	3.530	0.0176	11.608	0.9527	0.3302
39	**7.82**	1.7761	146.67	0.9791	3431	1716	2.649	0.0369	0.1328	4.722	0.0175	11.404	0.9531	0.3339
40	**8.85**	1.7310	148.34	0.9827	3331	1699	2.619	0.0411	0.1414	3.960	0.0182	12.458	0.9498	0.3689
41	**9.44**	1.7067	149.34	0.9848	3263	1683	2.603	0.0437	0.1460	4.253	0.0187	12.833	0.9441	0.4184
42	**8.60**	1.7257	148.50	0.9818	3323	1692	2.614	0.0417	0.1434	4.036	0.0183	12.551	0.9449	0.3388

43	**8.93**	1.7164	148.34	0.9838	3293	1695	2.606	0.0426	0.1449	4.138	0.0185	12.993	0.9286	0.3387
44	**11.28**	1.6523	150.73	0.9933	3077	1687	2.565	0.0498	0.1541	4.960	0.0198	14.466	0.8841	0.4937
45	**8.05**	1.7527	145.79	0.9835	3354	1736	2.625	0.0389	0.1366	3.704	0.0178	12.705	0.9537	0.2245
46	**8.07**	1.7668	145.39	0.9819	3384	1738	2.635	0.0343	0.1343	3.564	0.0176	12.636	0.9459	0.2572
47	**10.45**	1.7251	152.87	0.9851	3311	1633	2.640	0.0420	0.1387	4.019	0.0187	11.294	0.5037	0.0808
48	**7.72**	1.7937	149.10	0.9758	3497	1661	2.678	0.0355	0.1288	3.328	0.0171	9.567	0.6925	0.2630
49	**8.39**	1.6447	148.60	0.9828	3276	1671	2.536	0.0505	0.1678	5.184	0.0195	13.228	0.8812	0.1278
50	**9.25**	1.6218	149.67	0.9869	3201	1620	2.523	0.0536	0.1710	5.551	0.0198	13.861	0.9061	0.0856
51	**9.41**	1.6089	149.80	0.9883	3181	1665	2.513	0.0554	0.1741	5.785	0.0200	14.085	0.8867	0.0993
52	**9.56**	1.6210	149.86	0.9877	3187	1674	2.524	0.0538	0.1704	5.562	0.0198	13.825	0.9088	0.1299
53	**9.41**	1.6866	148.94	0.9847	3271	1674	2.581	0.0458	0.1527	4.528	0.0189	12.900	0.9416	0.2673
54	**8.81**	1.6988	148.80	0.9827	3315	1678	2.590	0.0445	0.1507	4.374	0.0186	12.279	0.9381	0.2340
55	**9.17**	1.6823	149.21	0.9846	3282	1672	2.578	0.0462	0.1541	4.596	0.0189	13.157	0.9276	0.2148
56	**8.48**	1.7106	148.37	0.9820	3320	1643	2.598	0.0431	0.1479	4.219	0.0186	12.681	0.9330	0.2496
57	**8.32**	1.7253	147.38	0.9807	3347	1665	2.606	0.0416	0.1450	4.045	0.0184	12.592	0.9419	0.1936
58	**9.17**	1.6600	149.07	0.9850	3248	1686	2.556	0.0487	0.1611	4.917	0.0192	13.856	0.9265	0.1639
59	**9.90**	1.6321	149.53	0.9881	3071	1596	2.534	0.0521	0.1661	5.335	0.0200	14.916	0.8914	0.2564
60	**7.96**	1.6797	148.13	0.9811	3342	1584	2.566	0.0458	0.1581	4.582	0.0184	12.913	0.9307	0.1837
61	**6.36**	1.7805	144.85	0.9748	3478	1663	2.638	0.0361	0.1342	3.426	0.0176	11.495	0.9390	0.1764
62	**7.24**	1.9741	145.44	0.9750	3560	1727	2.832	0.0235	0.0887	1.998	0.0145	9.647	0.9775	0.3138
63	**8.77**	1.6965	148.57	0.9830	3318	1682	2.587	0.0447	0.1516	4.410	0.0187	12.809	0.9285	0.2201
64	**8.98**	1.7133	149.70	0.9854	3307	1692	2.612	0.0429	0.1441	4.167	0.0184	12.567	0.9476	0.3277
65	**9.36**	1.6899	149.18	0.9846	3271	1670	2.585	0.0454	0.1516	4.482	0.0189	12.817	0.9349	0.3446
66	**9.57**	1.6837	149.29	0.9857	3253	1672	2.581	0.0461	0.1526	4.564	0.0190	12.981	0.9315	0.3116
67	**9.56**	1.7089	147.66	0.9829	3355	1686	2.593	0.0433	0.1494	4.253	0.0186	12.292	0.8753	0.3272
68	**8.78**	1.6783	149.23	0.9849	3283	1659	2.574	0.0467	0.1556	4.656	0.0189	13.035	0.9197	0.2393
69	**10.19**	1.6071	150.46	0.9903	3127	1650	2.516	0.0557	0.1719	5.792	0.0201	14.146	0.9055	0.1919
70	**13.11**	1.5010	154.61	1.0098	2706	1630	2.452	0.0733	0.1819	8.206	0.0221	17.218	0.7744	0.3029
71	**6.96**	1.7669	146.33	0.9766	3483	1732	2.636	0.0375	0.1370	3.572	0.0177	10.551	0.9549	0.1965
72	**6.87**	1.7650	146.33	0.9770	3479	1732	2.634	0.0377	0.1372	3.589	0.0178	10.728	0.9586	0.1975
73	**6.87**	1.7645	146.32	0.9770	3478	1731	2.634	0.0377	0.1375	3.594	0.0178	10.831	0.9586	0.1975
74	**8.31**	1.7246	147.77	0.9815	3371	1689	2.608	0.0416	0.1457	4.059	0.0182	12.360	0.9375	0.3130
75	**8.68**	1.7220	148.00	0.9822	3358	1691	2.607	0.0420	0.1457	4.089	0.0182	12.340	0.9432	0.3170
76	**8.48**	1.7240	147.88	0.9820	3361	1689	2.609	0.0417	0.1453	4.064	0.0182	12.362	0.9419	0.3215
77	**9.89**	1.6772	149.48	0.9873	3255	1655	2.577	0.0468	0.1543	4.650	0.0189	12.986	0.9461	0.2065
78a	**8.49**	1.7075	147.79	0.9823	3356	1665	2.592	0.0433	0.1501	4.266	0.0185	12.369	0.9441	0.2897
78b	**8.47**	1.7040	148.01	0.9825	3348	1662	2.590	0.0437	0.1508	4.311	0.0185	12.442	0.9448	0.2830
78c	**8.84**	1.7089	147.48	0.9826	3364	1667	2.592	0.0431	0.1500	4.246	0.0184	12.453	0.9363	0.2527
79	**9.27**	1.6923	148.55	0.9846	3322	1656	2.584	0.0450	0.1527	4.459	0.0187	12.777	0.9425	0.2256
80	**8.67**	1.6954	148.36	0.9834	3332	1663	2.585	0.0447	0.1524	4.421	0.0187	12.666	0.9446	0.2604
81	**8.86**	1.7156	148.29	0.9831	3335	1675	2.604	0.0426	0.1468	4.164	0.0184	12.506	0.9455	0.3295
82	**8.37**	1.7188	147.87	0.9818	3375	1696	2.603	0.0423	0.1475	4.142	0.0184	12.330	0.9424	0.3246
83	**10.46**	1.7012	148.18	0.9849	3315	1699	2.591	0.0440	0.1508	4.342	0.0186	12.787	0.8847	0.4286
84a	**11.73**	1.6763	149.27	0.9888	3233	1679	2.576	0.0468	0.1533	4.656	0.0192	13.453	0.8676	0.5240
84b	**11.78**	1.6727	149.23	0.9885	3257	1679	2.572	0.0471	0.1572	4.722	0.0193	13.198	0.8676	0.5434
85a	**10.28**	1.7001	147.63	0.9840	3339	1689	2.585	0.0440	0.1524	4.388	0.0188	12.649	0.8826	0.4368
85b	**10.71**	1.6974	148.18	0.9856	3307	1689	2.588	0.0444	0.1516	4.388	0.0187	12.659	0.8826	0.4500
86a	**10.29**	1.7038	148.12	0.9851	3309	1695	2.593	0.0437	0.1499	4.596	0.0186	12.638	0.8830	0.4501
86b	**10.66**	1.6814	148.79	0.9871	3263	1695	2.577	0.0461	0.1552	4.596	0.0191	13.202	0.8829	0.4408
87	**10.62**	1.6920	147.71	0.9850	3335	1687	2.579	0.0449	0.1543	4.481	0.0190	12.774	0.8839	0.4658
88	**10.64**	1.6753	147.43	0.9928	3245	1693	2.568	0.0468	0.1561	4.683	0.0192	13.563	0.8226	0.4534

89a	**10.56**	1.6981	148.18	0.9860	3305	1684	2.589	0.0443	0.1511	4.373	0.0184	12.687	0.8805	0.4735
89b	**10.40**	1.6799	148.29	0.9861	3305	1684	2.572	0.0462	0.1570	4.623	0.0192	13.321	0.8805	0.4507
90a	**10.89**	1.6755	148.86	0.9881	3259	1683	2.572	0.0467	0.1565	4.673	0.0192	13.235	0.8801	0.4642
90b	**10.15**	1.7009	147.69	0.9844	3337	1683	2.587	0.0439	0.1519	4.346	0.0188	12.541	0.8801	0.4614
91a	**10.61**	1.6923	147.76	0.9854	3331	1680	2.580	0.0449	0.1538	4.757	0.0190	12.674	0.8800	0.4888
91b	**10.98**	1.6702	148.41	0.9874	3287	1681	2.564	0.0473	0.1589	4.757	0.0194	13.354	0.8800	0.4806
92a	**10.45**	1.6747	148.74	0.9872	3275	1679	2.570	0.0474	0.1573	4.747	0.0193	13.349	0.8737	0.4897
92b	**10.77**	1.6699	149.23	0.9891	3236	1680	2.570	0.0467	0.1576	4.346	0.0194	13.541	0.8737	0.4969
93	**10.72**	1.6639	148.85	0.9885	3265	1674	2.561	0.0480	0.1601	4.842	0.0196	13.551	0.8736	0.5129
94	**8.82**	1.6942	148.24	0.9832	3336	1675	2.583	0.0448	0.1532	4.442	0.0187	13.021	0.9292	0.3012
95	**10.03**	1.6869	147.45	0.9871	3284	1673	2.574	0.0456	0.1522	4.504	0.0188	13.329	0.9387	0.3103
96	**9.07**	1.6904	148.33	0.9840	3333	1668	2.580	0.0452	0.1539	4.487	0.0188	13.116	0.9325	0.3155
97	**10.53**	1.6767	148.40	0.9856	3300	1684	2.569	0.0467	0.1573	4.677	0.0191	13.209	0.8716	0.4039
98	**7.59**	1.7054	147.05	0.9798	3410	1661	2.583	0.0432	0.1526	4.270	0.0182	12.454	0.9273	0.3439
99	**9.87**	1.6819	147.44	0.9828	3372	1681	2.565	0.0457	0.1579	4.569	0.0187	12.712	0.8701	0.4484
100a	**9.26**	1.6952	146.53	0.9844	3332	1645	2.574	0.0442	0.1518	4.361	0.0184	12.861	0.7395	0.3664
100b	**9.46**	1.6964	147.16	0.9813	3389	1645	2.576	0.0441	0.1549	4.892	0.0184	12.350	0.8696	0.4576
100c	**11.48**	1.6612	148.88	0.9879	3259	1682	2.558	0.0485	0.1601	4.380	0.0193	13.493	0.8696	0.3875
101a	**11.25**	1.6614	148.81	0.9881	3289	1680	2.558	0.0484	0.1613	4.899	0.0195	13.253	0.8711	0.5177
101b	**10.87**	1.6484	148.90	0.9882	3214	1657	2.546	0.0501	0.1631	5.092	0.0196	13.771	0.8711	0.4207
101c	**5.60**	1.7569	144.95	0.9755	3419	1719	2.616	0.0383	0.1409	3.687	0.0187	11.433	0.8811	0.0241
102	**5.47**	1.7568	145.06	0.9732	3474	1670	2.615	0.0382	0.1430	4.459	0.0181	11.177	0.9141	0.1634
103	**5.64**	2.1460	141.96	0.9723	3654	1769	2.973	0.0161	0.0628	1.279	0.0119	7.941	0.9901	0.2054
104	**5.82**	2.1172	143.32	0.9712	3652	1606	2.954	0.0169	0.0668	1.372	0.0122	8.500	0.9535	0.2181
105	**6.68**	2.0627	144.08	0.9728	3614	1735	2.907	0.0192	0.0745	1.578	0.0130	8.911	0.9596	0.1694
106	**7.34**	1.6954	147.48	0.9797	3393	1670	2.576	0.0449	0.1548	4.399	0.0184	12.669	0.9300	0.2855
107	**7.44**	1.7298	148.16	0.9803	3416	1636	2.613	0.0409	0.1437	3.962	0.0179	12.151	0.9388	0.4217
108	**7.55**	1.7000	147.82	0.9790	3405	1601	2.582	0.0430	0.1533	4.243	0.0179	12.266	0.8996	0.4117
109	**7.69**	1.7014	148.50	0.9806	3384	1609	2.588	0.0431	0.1514	4.240	0.0180	12.459	0.8846	0.3970
110	**8.24**	1.7339	148.33	0.9811	3368	1690	2.620	0.0408	0.1419	3.942	0.0181	12.072	0.9388	0.3098
111a	**8.25**	1.7690	149.15	0.9821	3390	1627	2.660	0.0374	0.1308	3.516	0.0177	11.748	0.9428	0.5286
111b	**5.11**	1.9900	120.32	0.9761	3604	1627	2.622	0.0264	0.1217	na	0.0260	6.984	na	na
112	**8.36**	1.7662	149.14	0.9823	3387	1683	2.658	0.0376	0.1314	3.545	0.0177	11.877	0.9442	0.5282
113	**8.69**	1.7323	148.48	0.9878	3367	1670	2.619	0.0410	0.1425	3.966	0.0182	12.211	0.9370	0.3850
114	**8.73**	1.7100	149.41	0.9843	3321	1680	2.606	0.0436	0.1461	4.216	0.0183	12.951	0.7878	0.3662
115	**8.75**	1.6876	149.12	0.9828	3354	1676	2.581	0.0451	0.1534	4.470	0.0186	12.826	0.9213	0.4523
116	**9.32**	1.7136	148.09	0.9831	3335	1694	2.601	0.0428	0.1479	4.199	0.0186	12.808	0.9331	0.3625
117	**9.32**	1.7098	149.97	0.9864	3286	1638	2.611	0.0434	0.1442	3.749	0.0185	12.763	0.9519	0.3469
118	**9.84**	1.7032	150.54	0.9881	3252	1694	2.609	0.0441	0.1445	4.279	0.0187	12.782	0.9436	0.3982
119a	**13.78**	1.4100	175.84	1.0261	2436	1556	2.435	0.0906	0.1697	11.132	0.0106	18.406	0.7443	na
119b	**15.41**	1.4690	152.02	1.0175	2745	1711	2.415	0.0790	0.1852	9.128	0.0225	16.928	0.7436	-0.2361
119c	**4.47**	2.0100	116.38	0.9695	3710	na	2.591	na	na	na	na	6.659	0.7443	na
120	**9.95**	1.6951	150.30	0.9874	3274	1689	2.599	0.0449	0.1479	4.394	0.0188	12.909	0.9412	0.3923
121	**11.09**	1.6562	151.40	0.9921	3182	1681	2.572	0.0494	0.1552	4.928	0.0193	13.649	0.9207	0.2990
122	**11.20**	1.6654	149.25	0.9887	3223	1678	2.565	0.0481	0.1565	4.819	0.0195	13.911	0.6915	0.4343
123a	**8.10**	1.7167	168.29	0.9839	3337	1660	2.688	0.0405	0.1323	3.737	0.0102	11.148	0.9019	-0.3924
123b	**9.86**	1.6490	150.34	0.9870	3222	1665	2.554	0.0501	0.1618	5.074	0.0194	13.625	0.9019	0.2039
123c	**17.18**	1.4970	153.57	1.0211	2609	1651	2.454	0.0743	0.1729	8.280	0.0224	17.733		0.5234
124	**4.94**	2.2842	175.28	0.9689	3705	1914	3.251	na	na	na	na	7.317	0.9484	0.4635
125	**4.89**	1.7831	157.66	0.9798	3521	1701	2.706	0.0341	0.1255	3.131	0.0153	9.171	0.9532	0.0308
126	**4.59**	1.8963	157.20	0.9708	3613	1768	2.816	0.0265	0.0980	2.281	0.0107	7.453	0.9717	-0.4006

127	**5.59**	1.8506	157.99	0.9731	3543	1751	2.777	0.0296	0.1068	8.382	0.0108	8.410	0.9709	-0.4569
128	**7.43**	1.6828	164.51	0.9758	3294	1724	2.636	0.0430	0.1484	4.114	0.0070	9.284	0.9418	0.1333
129	**9.62**	1.6765	172.41	0.9883	3255	1602	2.659	0.0443	0.1421	4.221	0.0109	11.112	0.7422	0.0742
70T	**21.89**	1.4279	155.56	1.0391	2430	1630	2.412	0.0891	0.1616	8.206	0.0236	19.130	-0.0444	0.6347
44T	**18.31**	1.5836	150.50	1.0090	2900	1705	2.511	0.0592	0.1658	6.135	0.0214	16.090	-0.2164	0.6316
83T	**19.32**	1.5773	152.28	1.0105	2966	1623	2.516	0.0600	0.1663	4.342	0.0209	15.439	0.5127	0.6217
97T A	**14.90**	1.6551	147.73	0.9982	3146	1613	2.555	0.0494	0.1567	4.954	0.0190	14.389	0.4200	0.5613
97T B	**22.10**	1.5190	152.29	1.0277	2617	1613	2.475	0.0494	0.1567	7.591	0.0195	14.389	0.1320	0.6706

na – not applicable, indicates that the value does not exist.

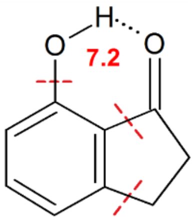

62

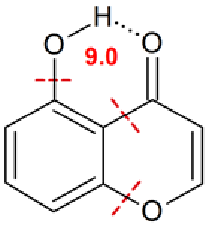

64

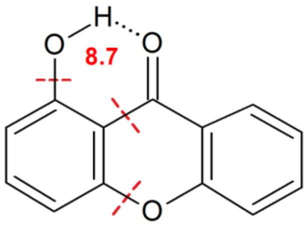

114

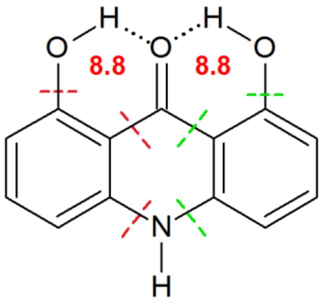

115

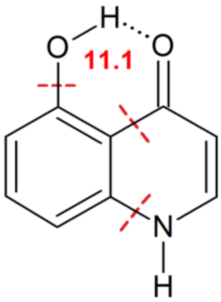

121

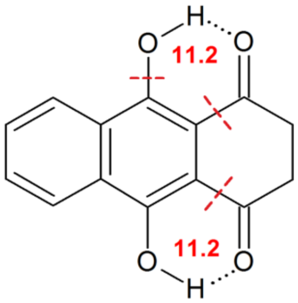

122

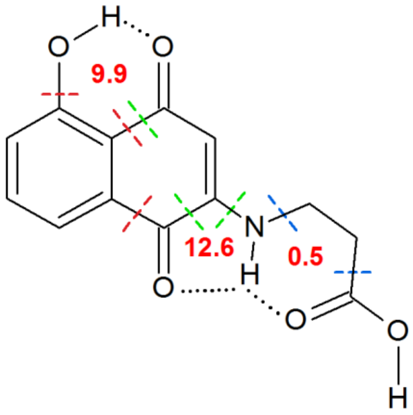

77

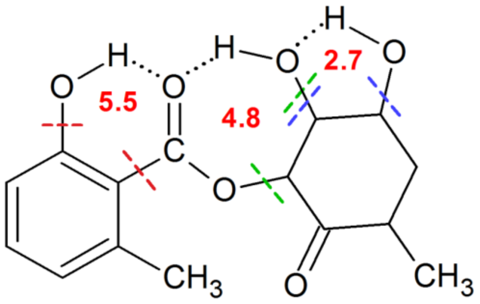

102

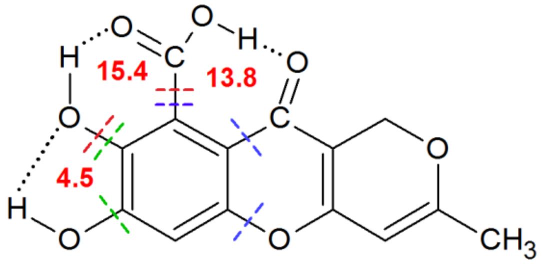

119

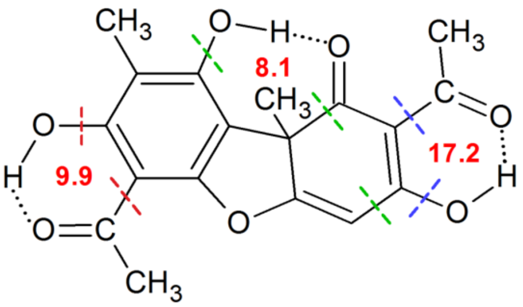

123

Figure S1. Examples of MTA fragmentation employed for selected structures analyzed in the study. Different colors represent different fragmentation of different intramolecular hydrogen bonds.

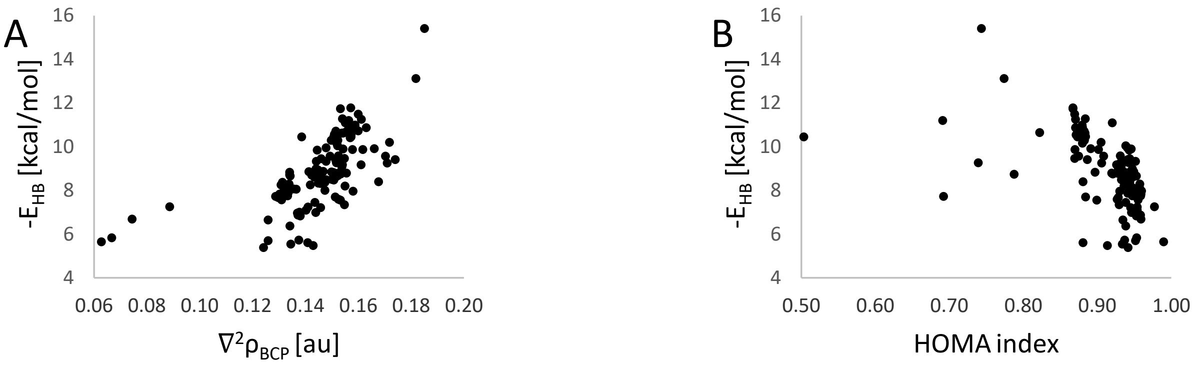

Figure S2. MTA Intramolecular Hydrogen Bond Energy [kcal/mol] as a Function of Laplacian of the Electron Density in the Bond Critical Point [au] (A) and its HOMA Index (B) for Structures with Phenolic Intramolecular Hydrogen Bonding.
